# Low-noise tunable deep-ultraviolet supercontinuum laser

**DOI:** 10.1038/s41598-020-75072-y

**Published:** 2020-10-28

**Authors:** Callum R. Smith, Asbjørn Moltke, Abubakar I. Adamu, Mattia Michieletto, Patrick Bowen, Peter M. Moselund, Christos Markos, Ole Bang

**Affiliations:** 1 DTU Fotonik, Technical University of Denmark, 2800 Kgs. Lyngby, Denmark; 2 NKT Photonics A/S, Blokken 84, 3460 Birkerød, Denmark; 3 NORBLIS IVS, Virumgade 35D, 2830 Virum, Denmark

**Keywords:** Optics and photonics, Physics

## Abstract

The realization of a table-top tunable deep-ultraviolet (UV) laser source with excellent noise properties would significantly benefit the scientific community, particularly within imaging and spectroscopic applications, where source noise has a crucial role. Here we provide a thorough characterization of the pulse-to-pulse relative intensity noise (RIN) of such a deep-UV source based on an argon (Ar)-filled anti-resonant hollow-core (AR HC) fiber. Suitable pump pulses are produced using a compact commercially available laser centered at 1030 nm with a pulse duration of 400 fs, followed by a nonlinear compression stage that generates pulses with 30 fs duration, 24.2 μJ energy at 100 kHz repetition rate and a RIN of < 1%. Pump pulses coupled into the AR HC fiber undergo extreme spectral broadening creating a supercontinuum, leading to efficient energy transfer to a phase-matched resonant dispersive wave (RDW) in the deep-UV spectral region. The center wavelength of the RDW could be tuned between 236 and 377 nm by adjusting the Ar pressure in a 140 mm length of fiber. Under optimal pump conditions the RIN properties were demonstrated to be exceptionally good, with a value as low as 1.9% at ~ 282 nm. The RIN is resolved spectrally for the pump pulses, the generated RDW and the broadband supercontinuum. These results constitute the first broadband RIN characterization of such a deep-UV source and provide a significant step forward towards a stable, compact and tunable laser source for applications in the deep-UV spectral region.

## Introduction

The importance of a compact wavelength tunable laser source, capable of delivering ultrashort UV pulses cannot be overemphasized. Furthermore, in order to maximize performance within an intended application, it is important that the pulse-to-pulse energy fluctuation, defined by the relative intensity noise (RIN), is as low as possible. The semiconductor industry would directly benefit from the advent of such technology, since the shorter wavelength radiation allows inspection of nanoscale periodic structures with improved sensitivity^1,2^, whilst the low RIN ensures high signal-to-noise ratio. Furthermore, the combination of high photon energy and ultrashort duration is attractive for materials processing, since this can provide effective energy coupling to precise regions of optical materials^3^. The inherently high photon energy of UV radiation is sufficient to liberate electrons from many materials, thus a stable and tunable UV source would be highly applicable to angle-resolved spectroscopy^4^. The compact nature of the desired laser would set it apart from large and expensive UV sources, such as synchrotrons^5^ and free-electron lasers^6^. Furthermore, the simplicity and tunability of the source would distinguish it from bulk crystal nonlinear frequency conversion systems^7^.

A promising approach to develop a tunable UV laser is to utilize and combine the emerging hollow core (HC) fiber technology with noble nonlinear gases. This has been achieved at multi-gigawatt peak powers using capillary fibers^8,9^. HC photonic-crystal fibers (PCFs)^10^ offer a viable option for lower peak power levels, and with improved loss properties they offer a more practical implementation than capillaries. HC PCFs can include fibers with guiding based on a photonic bandgap^11^, a Kagomé lattice^12^, or a negative curvature anti-resonant (AR) structure^13^. The latter two of these designs can provide low-loss transmission over a broad bandwidth, and a relatively low anomalous dispersion environment, which are very attractive attributes for ultrashort pulse compression and extreme nonlinear optics^14^. Combining a HC fiber with gas allows control over the guiding properties of the fiber. The dispersion profile can be tailored via the pressure, allowing smooth control of the ratio between the contributions from the filling gas (all normal) and the fiber geometry (all anomalous). Hence, it is possible to adjust the guiding properties of the fiber, including the zero-dispersion wavelength (ZDW) across the transmission band. Coupling a pump pulse of sufficient energy at an appropriate wavelength within the anomalous dispersion regime of the fiber will initiate soliton compression, resulting in extreme spectral broadening. A RDW can be generated if the spectral broadening reaches the phase matching frequency, defined as the frequency at which the propagation constants of the pump (higher-order soliton) and the RDW are equal in the direction of evolution^15^. Consequently, an efficient transfer of energy from the soliton to the RDW ensues. The phase-matching frequency occurs on the other side of the ZDW, therefore, the position of the RDW can be tuned by simply varying the gas pressure within the HC fiber.

This concept has been employed using a range of gas species to generate UV radiation with broadband guiding HC PCFs^16–22^. The majority of these experiments relied on a titanium-sapphire laser to provide the pump pulses, resulting in an inherently large footprint and low repetition rate of 1 kHz to achieve the required pulse energy. However, a few experiments have utilized the nonlinearly compressed output of ytterbium-based lasers, which significantly increases the repetition rate to MHz regime^21,22^, an important consideration for maximizing signal-to-noise ratio and average power in certain applications. Furthermore, the use of an ytterbium-doped gain medium paves the way to a more compact and rugged design, owing to the outstanding performance of these lasers at high repetition rates^23,24^. Interestingly, despite the impressive results achieved using this technique, the pulse-to-pulse stability of the generated UV radiation is only recently gaining attention. One study demonstrated a deep-UV RDW at 275 nm with a RIN of 33.3% measured in the time domain, however this poor performance was partially attributed to the high RIN of 5.5% of the 2.45 µm pump laser^25^. This high RIN value for the pump was a result of wavelength conversion from a low-noise titanium-sapphire laser (0.5% RIN) using an optical parametric amplifier. Another study quotes a RIN value of 0.4% measured in the frequency domain, for 1030 nm pulses compressed to the single-cycle regime using a two-stage AR HC fiber compression scheme^22^. However, this study did not involve RDW generation and the UV component of the spectrum is not specifically isolated and measured.

Unfortunately, there are numerous definitions of noise, requiring different measurement techniques. Thus it difficult to compare quoted noise values, especially when there is a lack of information regarding the definitions and techniques. Without a clear understanding of what is meant by noise, and how it is measured, little confidence can be given to a quoted value. The RIN can be investigated in the time domain, where a large number of pulses are incident on a photodiode. The voltage trace for each pulse is recorded on an oscilloscope, and under appropriate conditions, i.e. when the pulse duration is significantly shorter than the photodiode response time, the peak voltage is proportional to the pulse energy, thus the RIN can be determined by analyzing the defined peak voltage value for each pulse^26–28^. Note that in this instance the term RIN is a little misleading, since the optical intensity cannot be determined, however despite this misnomer the nomenclature is maintained. An advantage of the time domain approach is that a distribution of pulse energies can be obtained. This fact was exploited to confirm the presence of optical rogue waves using wavelength-to-time conversion, where the long wavelength edge of a generated supercontinuum was spectrally filtered and temporally stretched in a highly dispersive fiber^29^. The stretched pulses allow real-time observation of the pulse train on an oscilloscope. RIN can also be investigated in the frequency domain using an electrical spectrum analyzer^30–34^. An advantage of this technique is that noise sources can be identified based on their corresponding frequency, i.e. lower frequencies could indicate mechanical vibrations, which once identified can be minimized. In this investigation we measure the RIN in the time domain (details in Materials and Methods). Note that for several reasons, RIN measurements in the frequency domain can give lower values than measurements in the time domain, especially when the integration frequency range does not extend to the laser repetition rate.

Here, we develop a widely-tunable table-top deep-UV supercontinuum source and present an investigation into the RIN properties of the generated deep-UV emission in an Ar-filled AR HC fiber, demonstrating that a value as low as 1.9% can be achieved under appropriate pumping conditions. Suitable pump pulses were generated using a commercially available 1030 nm seed laser operating at 100 kHz combined with a compact nonlinear compression stage based on an air-filled AR HC fiber. The energy, duration and RIN of the pump pulses are fully characterized, and subsequently used to pump a second AR HC fiber within a gas-cell to generate a supercontinuum containing deep-UV radiation, which is characterized in terms of RIN, tunability, power and beam profile. A discussion on these results is presented, alongside corroborating studies from numerical simulations. The relatively high repetition rate and compact system format, combined with a detailed noise investigation, takes the concept of a tabletop, tunable deep-UV source closer to a commercial reality.

## Results

### Nonlinear pulse compression

**Figure 1:**
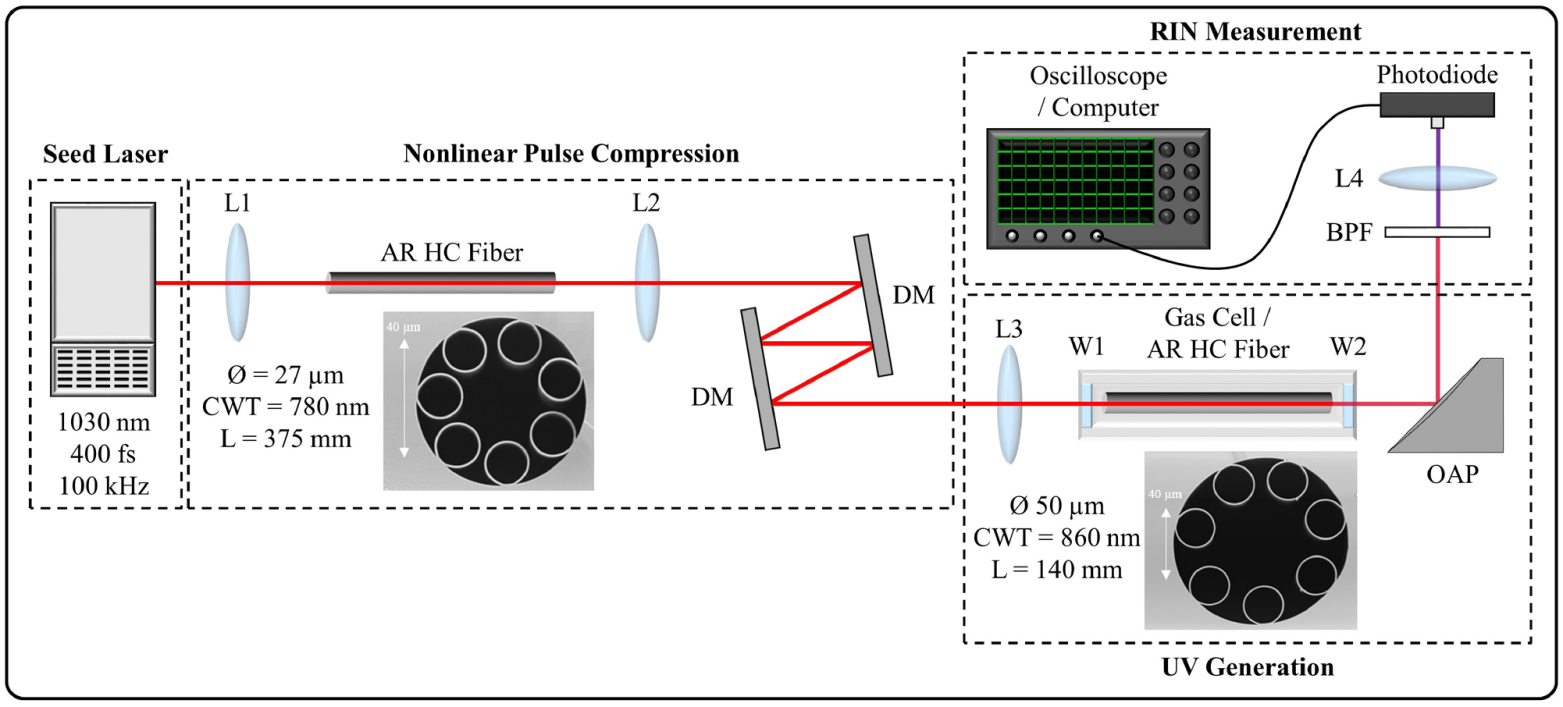
Experimental set-up: L1, L2, L3 = anti-reflection coated UV-fused silica plano-convex lens, L4 = uncoated calcium fluoride plano-convex lens, W1 = anti-reflection coated UV fused silica plane window, W2 = uncoated calcium fluoride plane window, DM = dispersive mirror, OAP = UV-enhanced aluminium off-axis parabolic mirror, BPF = spectral band-pass filter.

The deep-UV source developed in this investigation was seeded by the Origami 10XP (NKT Photonics A/S), operated at 100 kHz, producing pulses with a duration of 400 fs, energy of up to 35 µJ and a RIN of 0.5% at a center wavelength of 1030 nm. This pulse duration is too long to initiate the required soliton dynamics to achieve RDW generation in our experimental arrangement. Therefore, a nonlinear pulse compression stage was employed to reduce the initial pulse duration, as in^21^. This technique utilizes a Kerr medium, whereby the spectral bandwidth of the pulse is increased through self-phase modulation (SPM), creating a positive linear chirp across the central portion of the pulse. This chirp can be subsequently compensated by a linear dispersive element providing anomalous dispersion, leading to temporal compression. The first stage of this two-stage process requires a suitable nonlinear material that supports the peak powers involved (~ 80 MW) and allows for subsequent focusing into the UV-generation fiber. Conventional solid-core silica fibers are not suitable since the peak powers involved are significantly higher than the critical power for self-focusing (~ 4 MW)^35^, thus laser-induced damage is inevitable. There are examples of systems that have achieved compression of pulses with similar peak powers in non-guiding bulk media, however these systems have complex multi-pass^36^ or multi-component^37^ architectures, and suffer from inherent spatial distortions due to the non-guiding nature^38^. Despite gas having a significantly lower nonlinear index than silica, gas-filled HC fibers can provide high-quality modal guidance over long interaction lengths, thus allowing suitable nonlinear phase to accumulate. Additionally, these fibers offer a significantly higher self-focusing critical power^39^ and damage threshold compared to conventional solid-core fibers, thus HC gas-filled fiber is an attractive platform for pulse compression in this experiment. The compression system can be further simplified by operating without the use of a gas-cell, thus the fiber is simply filled with ambient air at atmospheric pressure, as in^40^. We employed this approach, utilizing a 375 mm length of AR HC fiber (provided by NKT Photonics A/S) with 27 µm core diameter and 780 nm capillary wall thickness (CWT). Note that this places the ZDW of the fiber at ~ 940 nm, and thus the fiber is pumped in the anomalous region, which is sub-optimal for pulse compression since it does not allow linearization of the chirp across the entire pulse. Nevertheless, effective pulse compression is achieved with this arrangement. A pair of identical plano-convex lenses with 50 mm focal length were used to couple light into the fiber and collimate light out of the fiber. Temporal compression was achieved with a pair of dispersive mirrors (HD59, Ultrafast Innovations GmbH). Each mirror reflection provides a group delay dispersion of ~ − 500 fs^2^, with a total of four reflections used in our system to give optimal compression at maximum available pulse energy. A schematic of the nonlinear compression stage is included in Fig. 1, with a SEM image of the fiber end-facet shown (inset).

Throughout this report: transmission efficiencies were recorded by measuring incident and transmitted average power with a thermal power meter, pulse durations were measured with an intensity autocorrelator, spectra were recorded using an integrating sphere fiber-coupled to a CCD spectrometer, and RIN was measured using a photodiode connected to an oscilloscope and computer (see Materials and Methods). The RIN measurement stage is shown after the UV generation stage in Fig. 1, however RIN measurements are also made after the seed laser and after the nonlinear compression stage with a similar arrangement.

**Figure 2:**
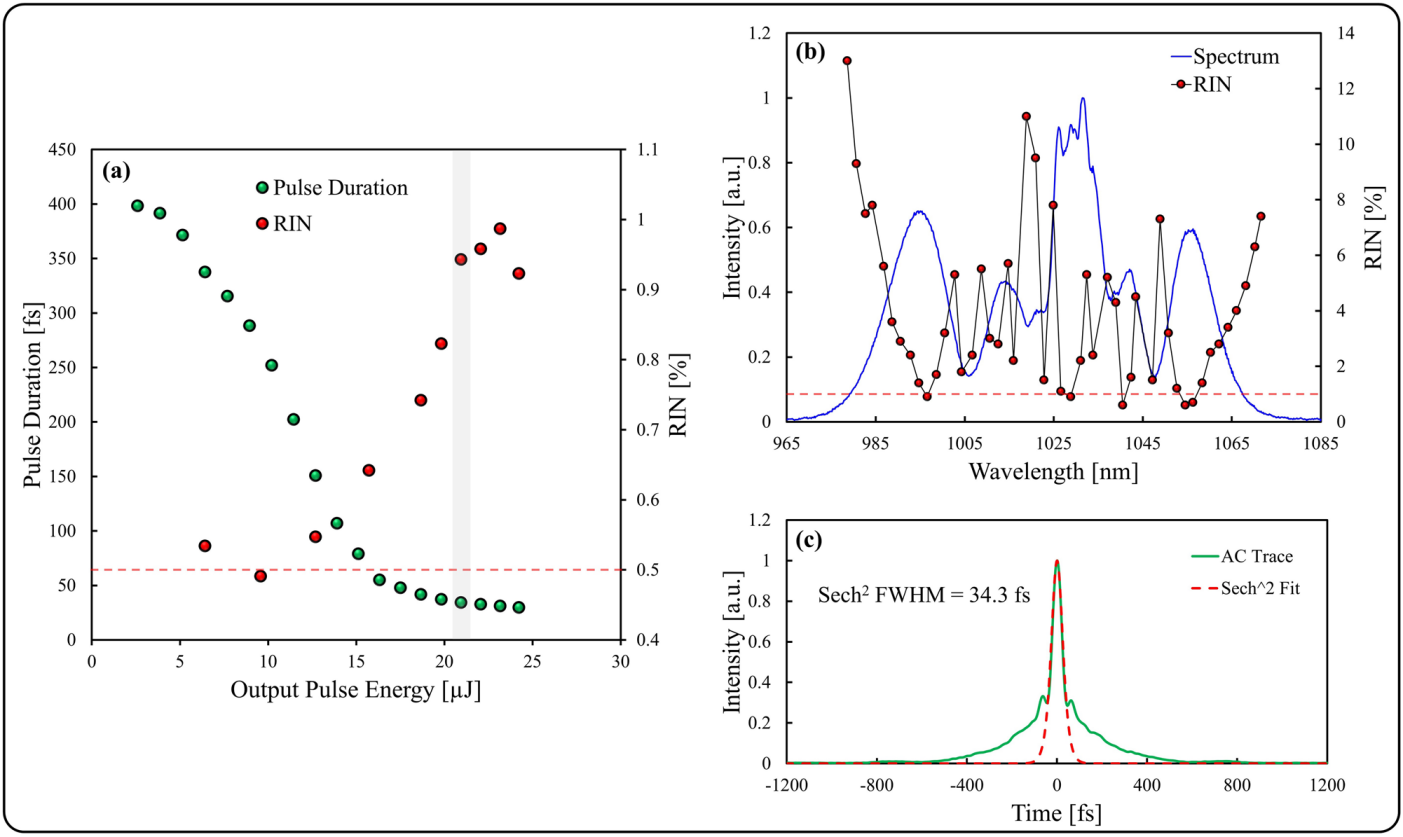
Characterization of pulse compression stage: (**a**) Pulse duration (green dots) and RIN (red dots) of compressed pulses as a function of output pulse energy, with the 0.5% RIN of the input seed indicated (dashed line), (**b**) Spectrum (blue line) and spectrally-resolved RIN (red connected dots) of the 20.9 µJ compressed output pulse, with the 0.94% RIN of the full pulse spectrum indicated (dashed line), (**c**) Intensity autocorrelation trace of the 20.9 µJ pulse (green line) and sech^2^-shaped pulse fitting.

The performance of the compression stage is summarized in Fig. 2. An overall transmission efficiency of ~ 69% was achieved through the entire nonlinear compression stage, providing a maximum output pulse energy of 24.2 µJ. The compressed pulse duration at the maximum pulse energy is 30 fs, corresponding to a compression factor of 13.3. The fiber length was chosen such that maximum pulse compression occurred at maximum available pulse energy, thus generating maximum peak power. The RIN of the compressed pulses tends to increase with pulse energy, however it is maintained at < 1% up to the maximum pulse energy of 24.2 µJ as shown in Fig. 2a, where the dashed line indicates the RIN of the seed laser. The compressed pulse at 20.9 µJ (gray shaded region in Fig. 2a) is further examined in Fig. 2b,c. The broadened pulse spectrum is shown in Fig. 2b, alongside the spectrally-resolved RIN, obtained using a monochromator to isolate the spectrum at ~ 2 nm increments with an ~ 3 nm full-width half-maximum (FWHM) bandwidth. The power spectral density has the expected features of SPM, with additional power distorting the profile between ~ 1020 and 1035 nm. This distortion is attributed to the excitation of higher-order modes or cladding light. This undesired light undergoes different spectral broadening and thus creates the observed spectral features. Figure 2c shows the intensity autocorrelation trace of the compressed pulse, with a sech^2^-shaped pulse profile fit to the central portion of the trace, indicating a FWHM of 34.3 fs. Despite measuring a total RIN of 0.94%, depicted by a dashed line in Fig. 2b, the RIN of the pulse varies considerably as a function of wavelength, within a range of 0.6–13% across the full spectrum. This rather large variation can be derived from the power fluctuations of the input pulse through fluctuations in the strength of the SPM. As the SPM induced spectral broadening is linear in power^41^, the center wavelength of each peak is jittering slightly. As a result hereof, the magnitude of the RIN value is correlated with the slope of the intensity, as demonstrated numerically in^42^. Thus, certain spectral components can exhibit significantly higher or lower RIN values compared to the total RIN, corresponding to the correlation within the pulse.

### Resonant dispersive wave generation

Figure 1 includes an SEM image of the fiber used in the UV generation stage. This fiber (provided by NKT Photonics A/S) was an AR HC fiber, with a 50 µm core diameter, 860 nm CWT and 140 mm length. The fiber was sealed in a gas-cell, which could be filled with Ar from 1 to 20 bar. Argon was selected since it is a noble gas and thus Raman-induced energy transfer and noise is avoided. The gas-cell was purged with Ar several times prior to experiments to minimize the presence of atmospheric air and impurities. Anti-reflection coated UV fused silica and uncoated calcium fluoride were used as input and output windows, respectively, allowing the laser beam to enter and exit the gas-cell. The pulses from the nonlinear compression stage are coupled into the fiber using a plano-convex lens with a focal length of 100 mm. The output beam is collimated using an aluminium-coated off-axis parabolic (OAP) mirror, employed to avoid chromatic aberrations, and sent on to the diagnostics. At the maximum input pulse energy of 24.2 µJ, the pulse energy at the output of the fiber was 16.9 µJ (70% transmission efficiency), measured by recording the average power after the OAP mirror and accounting for losses due to the gas-cell output window and the OAP mirror.

The generation of a RDW requires that the point of maximum compression is reached within the fiber. Thus, for fixed pump pulse parameters, this imposes upper and lower pressure limits for achieving RDW generation. Increasing the pressure within the fiber increases the nonlinearity and decreases the magnitude of the group velocity dispersion (GVD) at the pump wavelength, for pressures below the pressure at which the ZDW is equal to the pump. As the ZDW approaches the pump, the magnitude of the dispersion will become insufficient to allow compression within the available fiber length, and thus the upper pressure limit is reached. As the pressure is reduced, the lower nonlinearity will result in insufficient broadening from SPM within the available fiber length, and thus a lower pressure limit is reached. Additionally, it is well known that the RDW wavelength redshifts with pressure within these pressure limits^16^.

**Figure 3:**
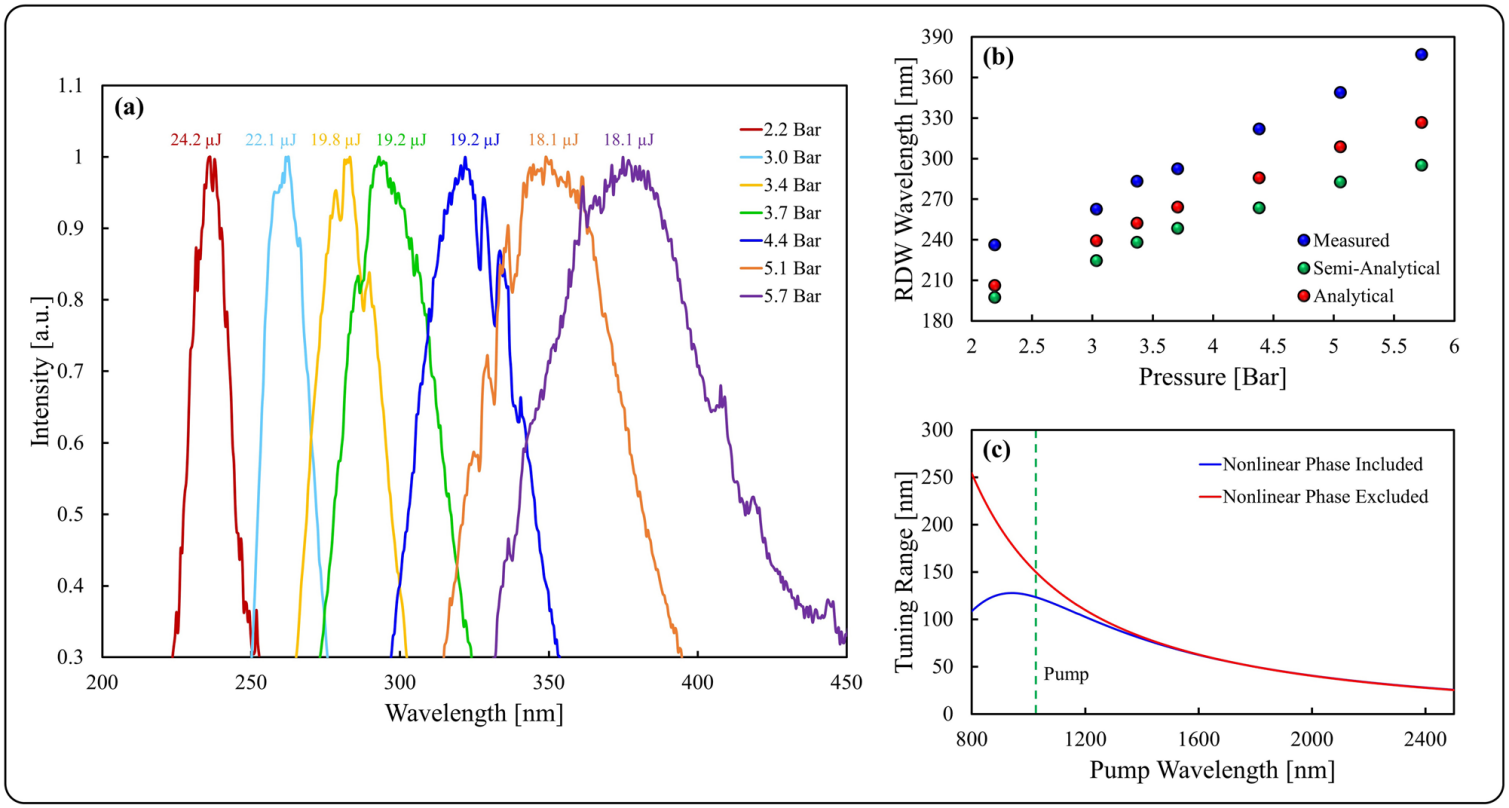
Tunability of the RDW with Ar pressure: (**a**) Normalized intensity spectrum of the RDW generated at various Ar pressures for optimal pump energy, (**b**) Measured RDW center wavelength as a function of Ar pressure for optimal pulse energy (blue dots) and predicted RDW center wavelength based on semi-analytical (green dots) and an analytical solution (red dots), (**c**) Predicted maximum tuning range of the RDW as a function of pump wavelength for a fixed duration (35 fs) and pulse energy (18 μJ) within the available pressure range (2.2–5.7 bar).

In Fig. 3a we show experimentally that the position of the RDW can be tuned with pressure and pump energy. It is well known that the temporal quality of soliton compression depends on the soliton number, with an optimum occurring for a soliton number around 4^43^. Therefore, for each pressure the pump pulse energy was tuned to an optimum value, provided above each spectrum in Fig. 3a. For the available fiber length of 140 mm the lower limit of pressure below which an RDW could not be generated with the available pulse parameters was 2.2 bar. At this pressure the optimal pulse energy was the maximum available of 24.2 µJ (30 fs duration), resulting in clean RDW generation at 236 nm. Increasing the pressure to 5.7 bar, we observed tunability of the RDW to 377 nm for an optimal pulse energy of 18.1 µJ (45 fs duration). This tuning range of 141 nm represents, to the best of our knowledge, the largest experimentally reported RDW tuning range for an Ar-filled HC fiber. Above 5.7 bar, clear RDW generation was not observed, as the RDW became obscured by other spectral features. Figure 3b depicts the measured RDW center wavelength as a function of pressure taken from the data presented in Fig. 3a. To confirm and explain the large tuning range, we consider theoretical predictions of the RDW center wavelength. In order to achieve RDW generation, the phase-mismatch, Δ*β*, between the propagation constant of the RDW, β_RDW_, and the nonlinear propagation constant of the pump, β_sol_, should be zero, described by: $\Delta \beta = \beta_{RDW} \left( {\omega_{RDW} } \right) - \beta_{sol} \left( {\omega_{RDW} } \right) = 0$, where *ω*_*RDW*_ represents the frequency of the RDW. Figure 3b shows predictions of the RDW based on two different estimates of β_sol_. The first is a semi-analytical prediction, which estimates $\beta_{sol} \left( {\omega_{RDW} } \right) \approx 2.3\gamma P_{0} N$, where γ is the nonlinear coefficient, *P*_*0*_ is the peak power of the pump, *N* is the soliton number, which in this case increases from ~ 3 to 6 as the pressure is increased from 2.2 to 5.7 bar, and the factor 2.3* N* is a result of numerical simulations as discussed in^44^. The second prediction is purely analytical, based on a solution for a higher-order soliton^25^, and estimates $\beta_{sol} \left( {\omega_{RDW} } \right) \approx \left( {2N - 1} \right)^{2} \left| {\beta_{2} } \right| / 2T_{0}^{2}$, where β_2_ is the GVD at the pump wavelength and *T*_*0*_ is the pump pulse duration. Note, that for this range of *N*, the approach based on an analytical solution for a higher-order soliton^25^ gives a closer prediction. One reason for this impressive tuning range is attributed to the choice of pump wavelength, 1030 nm, which is longer compared to titanium-sapphire lasers emitting at ~ 800 nm. To demonstrate this we used the more accurate model for RDW center wavelength prediction described above and calculated dispersion from the modified capillary model^45^, to plot the predicted maximum tuning range of the RDW as a function of pump wavelength, as shown in Fig. 3c with (blue line) and without (red line) the nonlinear phase contribution. Here we observe that it is important to include the nonlinear contribution below ~ 1.5 um, and the theory confirms an increase in tuning range by 13.4% going from 800 to 1030 nm. Interestingly, the theory predicts this tuning range could be even further increased by pumping at 940 nm. Furthermore, fiber design can be optimized to maximize the available tuning range.

### RIN investigation

**Figure 4:**
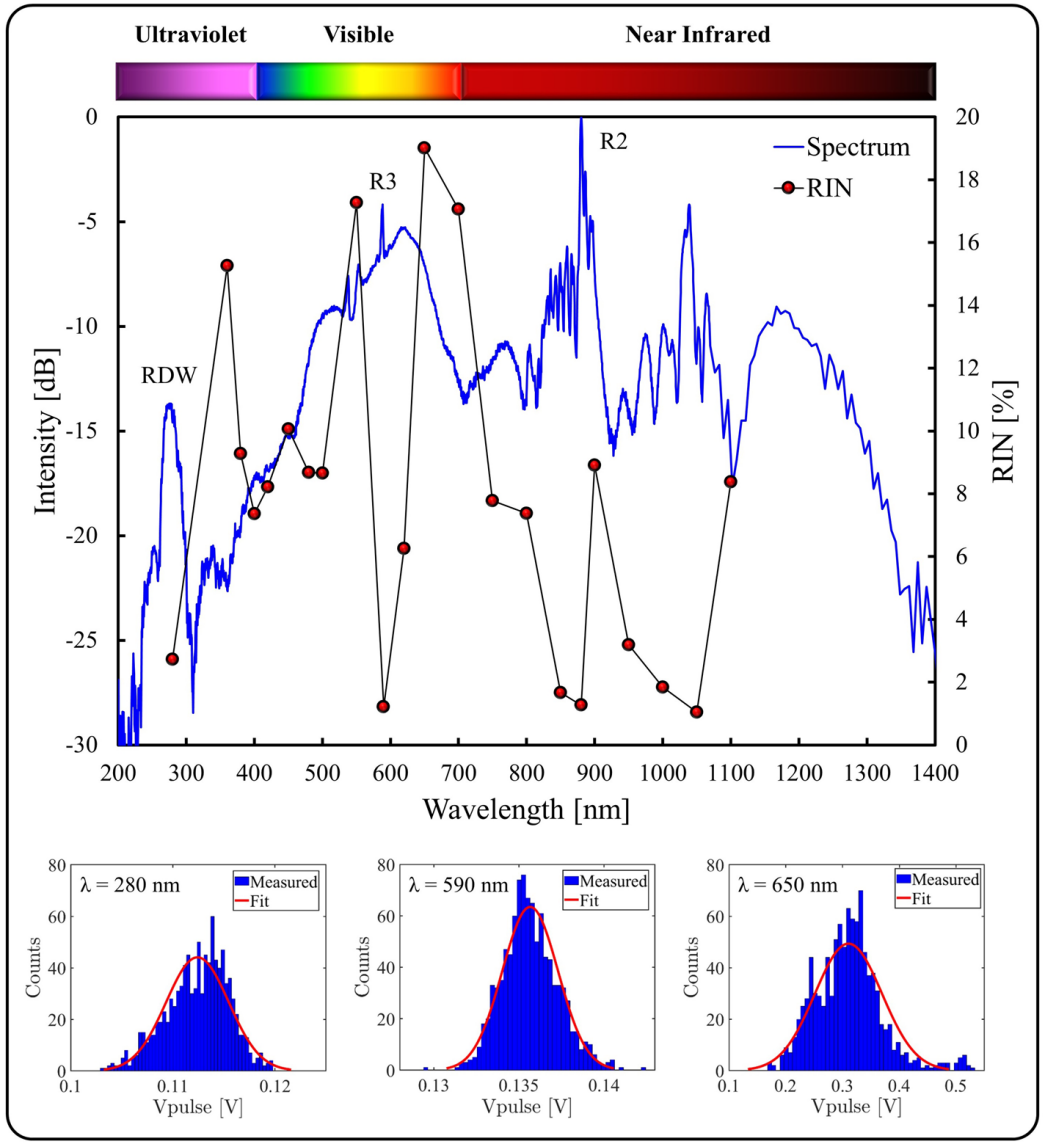
Spectral and RIN data for the generated supercontinuum: (Top) Spectrum generated for 20.9 µJ pulses at 3.4 bar of Ar (blue curve) with spectrally-resolved RIN (connected red dots), (Bottom) A sample of histogram plots showing the detected peak voltage distribution.

**Figure 5:**
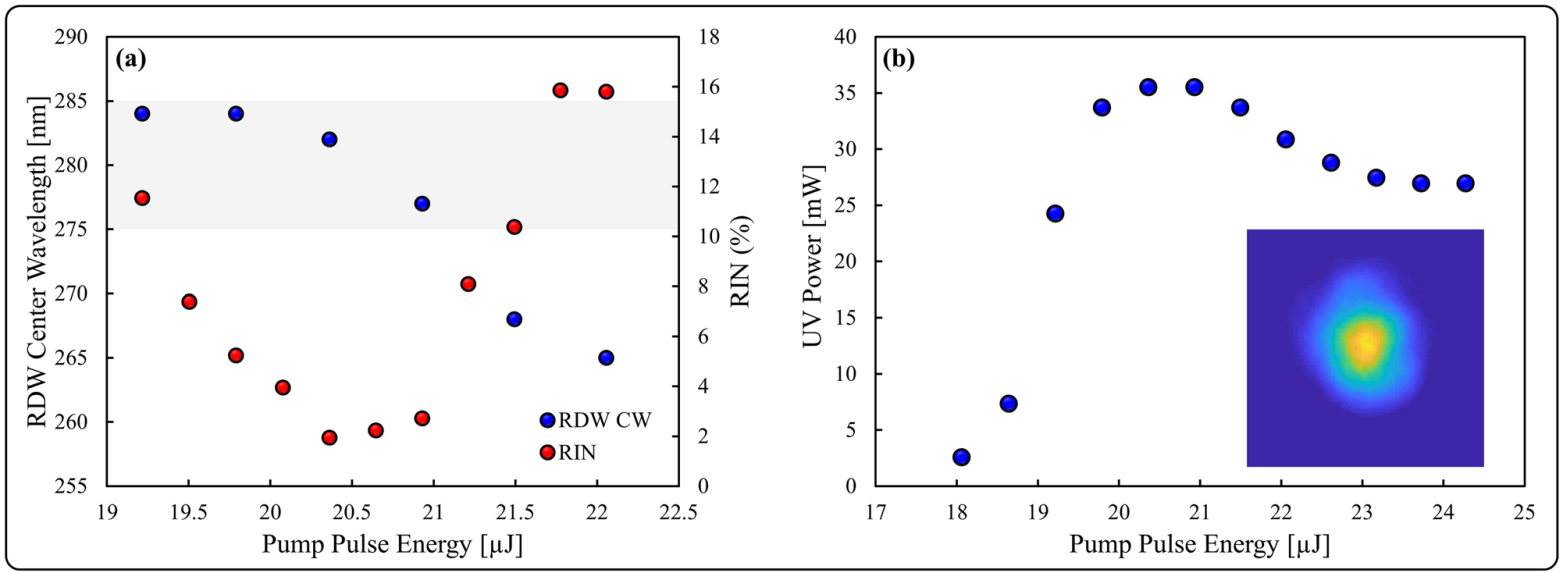
Characterization of UV radiation: (**a**) RIN and RDW center wavelength (RDW CW) as a function of pump energy, with the FWHM of spectral filter the shown (shaded gray), (**b**) UV power below 400 nm as a function of pump energy with far-field beam profile (inset).

To measure the RIN of the RDW, we used a spectral bandpass filter centered on 280 nm with a FWHM of 10 nm. We selected this bandpass filter since we could generate a RDW at this wavelength under stable conditions, while still enabling the investigation of the energy dependence. As shown in Fig. 3a, RDW generation could be achieved below 280 nm, however this requires the maximum available pump energy, and therefore would not allow us to investigate the impact of increasing pump pulse energy on the RDW RIN (as investigated in Fig. 5). To tune the RDW to match the spectral filter, the Ar pressure was adjusted to 3.4 bar and the pump parameters were set at 20.9 µJ energy and 34.3 fs duration, i.e. the parameters presented in Fig. 2b,c. This resulted in a RDW center wavelength of 277 nm with a FWHM of ~ 20 nm. The full spectrum generated under these conditions is shown in Fig. 4. Here we observe peaks resulting from the resonances dictated by the CWT at 880 nm and 588 nm, labelled R2 and R3 since they correspond to the second and third resonance respectively. Furthermore, we observe a clear RDW at 277 nm, albeit with a reduced power spectral density compared to other features of the spectrum. This reduced power spectral density can be partially attributed to the increased losses of the spectral detection system for deep-UV radiation, arising from the barium sulfate internal surface of the integrating sphere, the connecting fiber and the spectrometer. The spectral bandpass filter was employed to reject all wavelengths outside the transmission band, allowing direct access to the central frequency components of the RDW. This deep-UV radiation was focused onto the photodiode using an uncoated calcium fluoride lens with 50 mm focal length. The RIN of this radiation was measured to be 2.7%. Additionally, this measurement was repeated at different wavelengths using 10 nm FWHM spectral bandpass filters across the spectral bandwidth, as shown in Fig. 4. There are four distinct regions where low RIN, i.e. < 3%, was observed: the RDW, R2, R3 and the 1000–1050 nm band, corresponding to the wavelengths present in the pump pulse. Outside these regions the RIN is generally higher, with a value exceeding 19% at 650 nm. The histogram plots shown in Fig. 4 show the noise measurements conform reasonably to a normal distribution, with examples shown for 280 nm, 590 nm and 650 nm, which have RIN values of 2.72%, 1.21%, and 19.01% respectively.

The RIN of the RDW centered around 280 nm was further investigated by varying the pump pulse parameters. The pump energy threshold required to generate a RDW at 3.4 bar of Ar within the available length of fiber was 19.2 µJ, with the onset of RDW distortion occurring above 22.1 µJ, thus the RIN properties were investigated in this energy range. The center wavelength of the RDW blueshifts as the pump energy is increased over this range, as observed in Fig. 5a, which also shows the FWHM transmission band of the 280 nm spectral filter (shaded gray). The evolution of power in the entire UV region, i.e. < 400 nm, was measured by spatially separating frequency components using a calcium fluoride prism, allowing UV radiation to be isolated and directed to a thermal power meter. As observed in Fig. 5b, the power initially increases rapidly, reaching a maximum level of 35.4 mW between 20.4 and 20.9 µJ, before decreasing slightly and plateauing at higher energies. This plateau is attributed to an increasingly unsmooth pulse self-compression in the UV generation fiber, and consequently less efficient energy transfer to the RDW as the pump energy is increased. This power measurement level accounts for losses of the gas-cell output window, the OAP mirror and the prism. Therefore, 2.1% of the output power is contained within the UV, with an estimated 42% of that UV power contained in the RDW, which has an average power of ~ 15 mW, and a pulse energy of ~ 150 nJ. Considering the entire system of pulse compression and UV generation, the total conversion efficiency from the 1030 nm seed laser to the RDW at ~ 280 nm is 0.5%. Figure 5a also shows the RIN of the radiation transmitted by the spectral bandpass filter, which at threshold is measured at 11.5%. Initially, increasing the pump energy results in a decreasing RIN, and a minimum value of 1.9% is reached at 20.4 µJ, after which a further increase in pump energy leads to an increasing RIN, up to a value of 15.8% at 22.1 µJ. The high RIN measured at the lowest pump energy can be explained by the fact that the RDW process is close to threshold conditions, and thus small changes in pump conditions can have a pronounced effect on the RDW generation process. The subsequent decrease in RIN is attributed to two phenomena. Firstly, the energy in the RDW increases, as displayed in Fig. 5b, thus the impact of fluctuations on the RIN is reduced. Secondly, the wavelength of the RDW is shifted into the center of the filter, providing increased spectral overlap. The increase in RIN at higher pump pulse energies can be attributed to the fact that the RDW center wavelength shifts out of the FWHM transmission band of the 280 nm spectral filter. Thus, the sampled radiation is not generated from the central section of the RDW but actually from the wings and background UV radiation, which does not share the low noise properties of the RDW. As observed in Fig. 4, the RIN increases rapidly as the wavelength is increased from the RDW central wavelength. Therefore, it is shown that a careful choice of pump conditions is necessary to achieve pronounced RDW generation with noise properties considerably lower than the majority of the generated spectra. The beam was profiled in the far-field after the 280 nm spectral filter using a CMOS image sensor to acquire the image shown in Fig. 5 (b, inset), taken for a pump pulse energy of 20.4 µJ. The beam displays a reasonably Gaussian profile, indicating excitation of the fundamental mode, which is desirable in most applications.

### Numerical investigation

**Figure 6:**
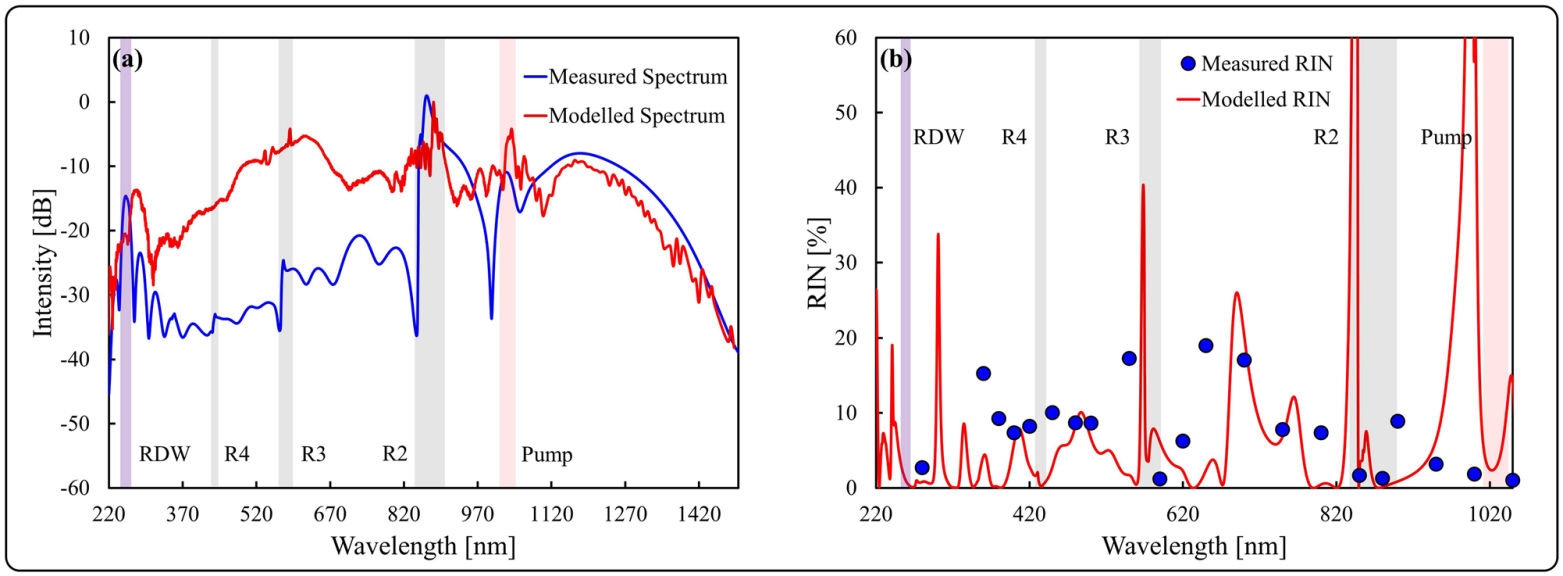
Numerically modelled and experimental obtained spectra and RIN at 3.4 bar Ar: (**a**) Measured spectrum for pumping with 20.9 µJ (blue line) and numerically modelled spectrum assuming 20 µJ pump energy (red line), (**b**) Measured RIN for pumping with 20.9 µJ (blue dots) and numerically modelled RIN assuming 20 µJ pump energy (red line). The following regions are indicated: pump (shaded red), R2, R3 and R4 (shaded gray) and the RDW (shaded purple).

Numerical modelling was employed to confirm the low RIN measured at the RDW and the resonant peaks. The supercontinuum generation process was simulated using the model described by Habib et al.^46,47^, which incorporates ionization using the Ammosov, Delone and Krainov model^48^. The RIN model incorporated amplitude and anti-correlated pulse duration noise of 1% and one photon per mode quantum noise^42^. The modelling assumed a bandwidth-limited sech^2^-shaped input pulse, with 13.6 µJ pulse energy and 35 fs pulse duration. Figure 6a shows the simulated and measured spectra between 220 and 1500 nm. Furthermore, the modelled FWHM of the pump radiation (shaded red), the fiber resonances dictated by the CWT at 866 nm (R2), 575 nm (R3) and 436 nm (R4) (shaded gray) and the RDW at 254 nm (shaded purple) are included. Whilst we note discrepancies between the magnitude of the intensity between the measured and simulated spectra, particularly in the visible region, we observe excellent agreement in the overall spectral bandwidth and shape and in the positions of the RDW and the resonant peaks, indicating the developed model is consistent at predicting the positions of these important features. The discrepancy in the visible spectrum primarily shows the shortcomings of the available analytical descriptions of resonances, which are more pronounced as the pump approaches R2. Figure 6b shows the simulated and measured RIN values between 220 and 1050 nm, again with the same shaded regions indicated. We firstly note that the magnitude of simulated and measured RIN values vary across a similar range, except for narrow discontinuities near the resonances. We observe that the simulated RIN drops below 4% in the vicinity of the resonances, the pump and the RDW. This trend is also observed empirically for R2, R3, the pump and the RDW. Note we did not measure an especially low RIN value at R4, attributed to suboptimal spectral overlap with our closest available filter at 450 nm, thus we may be sampling the spectral edge of the peak at R4. Additionally, the power observed at R4 was considerably lower than at R2 and R3, and not significantly higher than the surrounding spectrum. Thus, the low noise properties of R4 could be hidden by the higher noise of the background spectrum. Due to the decreasing impact at shorter wavelengths, resonances below R4 were not considered. Whilst there are slight discrepancies in the absolute values, the simulated regions of low RIN tend to agree with the experimental measurements.

## Discussion

The early development of deep-UV supercontinuum laser sources based on gas-filled HC fibers have been using < 50 fs pulses from large footprint titanium-sapphire pump lasers with repetition rates around 1 kHz. There has been little focus on the noise properties, except for a recent work reporting good long-term stability, but high pulse-to-pulse RIN values of 33.3% of a 275 nm RDW, attributed to the large RIN of 5.5% of the 2.45 µm pump^25^. The technology is now maturing to a level ready for applications, such as imaging and spectroscopy, which has seen a recent push towards table-top ytterbium-based lasers with MHz repetition rates as pump lasers, giving few-hundred fs pulses at center wavelengths of 1030 nm^21,22^. To get the necessary short pump pulses external pulse compression with chirped mirrors and a second gas-filled HC fiber is used the generate < 30 fs pump pulses^21,22^. With the push towards MHz and applications it has become even more important to assure low levels of RIN of the source and one could fear that the external pulse compression module could significantly add to the RIN of an otherwise low-noise seed laser. The issue about RIN and in particular the role of the compression module, has been the key motivation behind this work.

Here, we firstly demonstrated a compression module using an air-filled HC fiber (i.e. no gas-cell) that could give the necessary compression to around 30 fs. At maximum compression we achieved a pulse duration of 30 fs for 24.2 µJ pulses at 100 kHz repetition rate with 0.94% RIN. This corresponds to an addition of only 0.44% to the low-noise seed, which had a RIN of 0.5%. A thorough spectrally resolved measurement of the RIN of the compressed pulse revealed a RIN profile that was strongly oscillating between 0.6 – 13% around the spectrally averaged value of 0.94%. This implies that the pump noise can be further reduced by better control of the compression. Using pump pulses with a range of energies between 18.1 and 24.2 µJ and a range of pulse durations between 30 and 45 fs, we have demonstrated a wide tunability of the RDW center wavelength from 236 to 377 nm. This wide tuning range of over 140 nm is, to the best of our knowledge, the largest achieved using an Ar-filled HC fiber. Furthermore, under optimal conditions the RDW could be generated at 280 nm with a RIN value of only 1.9%. This is significantly lower than most commercially available supercontinuum source in the visible and near-infrared and documents that the tunable RDW technology is ready for applications. We also presented the first broadband measurement of the RIN profile of the gas-filled AR HC fiber based UV supercontinuum source and demonstrated that the RIN is strongly oscillating because of the spectral dips implicit for such a source, but stayed low around the RDW and the resonant peaks around 880 nm and 588 nm, as well as around the pump in the 1000–1050 nm band. The RDW and resonance wavelengths and the associated low levels of RIN was confirmed by numerical simulations.

Our study presents the first broadband characterization of the noise of these promising deep-UV supercontinuum sources. We have revealed that while the tunable RDW part of the spectrum has low noise and is thus ready for applications, the broadband source needs further development if it is to be used in imaging and spectroscopy applications requiring low noise over a broad bandwidth, such as in scatterometry-based imaging of nanostructures on semiconductor chips.

## Materials and methods

### RIN measurements

**Figure 7:**
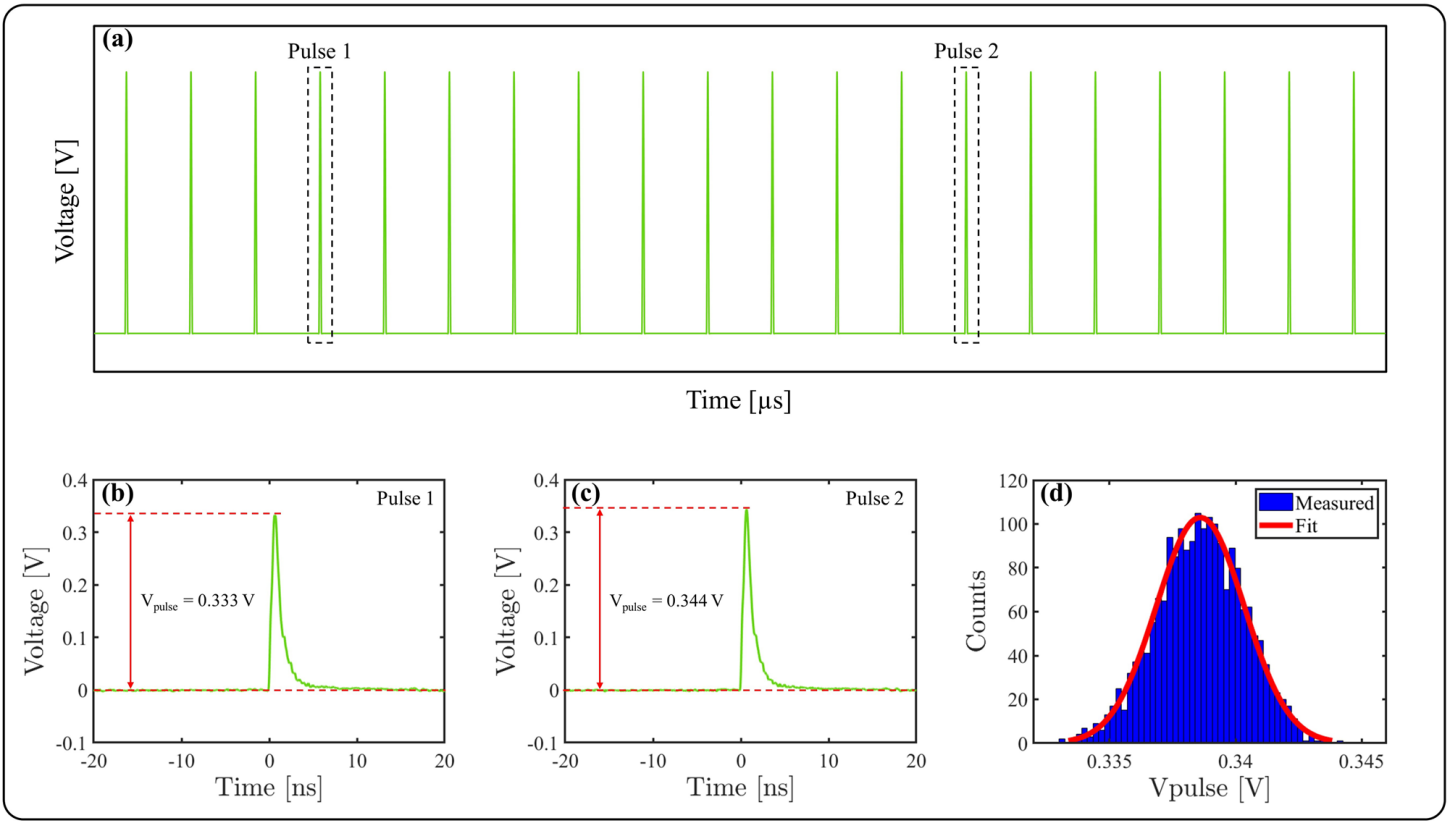
RIN measurement details: (**a**) Schematic of recorded pulse train, (**b**) example voltage response for pulse 1, (**c**) example voltage response for pulse 2, (**d**) example histogram of *V*_*pulse*_ for the Origami 10XP operating at 35 µJ.

The RIN measurements were performed using a fast silicon photodiode (DET10A2, Thorlabs, Inc.) connected to an oscilloscope (HDO9404, Teledyne LeCroy, Inc.). This is shown after the UV generation stage in Fig. 1; however note that the RIN measurement was also performed after the seed laser and the nonlinear compression stage. When taking RIN measurements, it is important to attenuate the laser power to a level suitable for the photodiode, and the method of attenuation varies depending on the measurement. For RIN measurements after the seed laser and after the nonlinear compression stage, i.e. those presented in Fig. 2a, the laser beam was attenuated by taking a reflection off an uncoated wedge at close to normal incidence, and subsequently further attenuated by transmission through absorptive neutral density filters. The initial wedge reflection is necessary to decrease the power to an appropriate level not to damage the absorptive neutral density filters. For the spectrally-resolved RIN measurement of the pump pulse, i.e. those presented in Fig. 2b, the beam was steered to a grating based monochromator (SP-2300i, Princeton Instruments, Inc.) using protected silver mirrors. By adjusting the grating angle and the width of an output slit, the wavelength and bandwidth of the transmitted radiation can be controlled. The FWHM was maintained at 3 nm throughout the measurements. The power of the transmitted radiation is suitable for direct attenuation with absorptive neutral density filters. Finally, for the spectrally-resolved RIN measurements of the generated supercontinuum, i.e. those presented in Fig. 4, the radiation was spectrally filtered by a bandpass filter and then directly attenuated using absorptive neutral density filters. Following attenuation, the laser beam is focused onto the detector, so that its energy falls entirely within the active area. We have found that RIN varies significantly across the spatial profile of the beam, therefore it is important to ensure the whole beam area is sampled. The photodiode generates a photocurrent in proportion to the optical energy on the detector, which is consequently converted into a voltage and displayed on the oscilloscope. Thus, the pulse train creates a series of voltage peaks at the laser repetition frequency, as shown schematically in Fig. 7a. The rise time of the photodiode is ~ 1 ns and thus several orders of magnitude greater than the pulse durations being considered in this investigation. Therefore, the duration of the voltage response to each pulse is a characteristic of the photodiode. The oscilloscope was operated at a sampling rate of 40 GS/s, corresponding to a sampling period of 25 ps, which was sufficiently short to avoid under sampling of the pulse profile. It is important that each pulse is clearly distinguishable from the next, and therefore the repetition rate must be suitably low such that adjacent pulses do not interfere. Furthermore, we ensured all our measurements were conducted in the linear response regime of the photodiode, a necessity for these measurements. To calculate RIN we require the voltage measurement of a sample of pulses. The oscilloscope runs a MATLAB script to identify an individual pulse and record the corresponding voltage response. In the pulse train schematic in Fig. 7a the first identified pulse is labeled pulse 1, for which an example of an actual recorded voltage response is shown in Fig. 7b. Once this process is complete the script finds a pulse, labeled pulse 2, and again records the voltage response, as shown for another example recorded voltage response in Fig. 7c. Note that pulses that reach the detector whilst the script is identifying and recording the voltage response of an individual pulse are not recorded. This is depicted schematically in Fig. 7a, where we see pulse 1 and pulse 2 are separated by several unrecorded pulses. This process continues until a pre-determined number of pulses have been recorded. A voltage value is then assigned to each pulse, given by *V*_*pulse*_, which under the appropriate conditions is proportional to the energy of the pulse. *V*_*pulse*_ is calculated by taking the difference in voltage between the peak of the pulse and the floor of the pulse, as indicated in Fig. 7b,c. Thus, an array of voltages is obtained, proportional to the energy of each pulse. A typical histogram showing the distribution of *V*_*pulse*_ for 2,000 pulses is shown in Fig. 7d. Here we can see that the data conforms to a normal distribution. The example data given in Fig. 7b–d is taken from the measurement of the output of the Origami 10XP operating at 35 µJ, i.e. the seed laser. The RIN is calculated according to:

1 $$ RIN = 100 \times \frac{\sigma }{\mu } $$

where *σ* is the standard deviation and *µ* is the mean of the array of *V*_*pulse*_. Since *V*_*pulse*_ is proportional to pulse energy, the RIN of *V*_*pulse*_ will be equal to the RIN of the pulse energies. Thus, the RIN defines the percentage change in energy (positive or negative) compared to the mean that will contain one standard deviation, which for a normal distribution corresponds to 34.1% of the data. The calculated RIN from the example of the Origami 10XP seed laser given in Fig. 7 is 0.5%. We maintain this definition and technique for RIN measurements presented throughout this paper.

### Spectral measurements

For spectral measurements the laser beam was directed into an integrating sphere (IC2, StellarNet, Inc.). The integrating sphere was fiber-coupled (QP600-1-SR-BX, Ocean Optics, Inc.) to a CCD-based spectrometer. For measurements in the range 200–1070 nm the HR2000 + (Ocean Optics, Inc.) spectrometer was used, and for measurements in the range 1070–1400 nm the NIRQuest (Ocean Optics, Inc.) spectrometer was used. The spectrum shown in Fig. 4 was joined together by matching the amplitude of the spectra obtained for the wavelength range around 1070 nm. All spectra are recorded over integration times significantly longer than the pulse period, and thus are averaged over a large number of pulses.

### Pulse duration measurements

Pulse durations were measured with an intensity autocorrelator (pulseCheck 50 USB, A.P.E. GmbH). A sech^2^-shaped pulse profile was assumed in order to provide a measure of the pulse duration.
